# RhizoPot platform: A high-throughput *in situ* root phenotyping platform with integrated hardware and software

**DOI:** 10.3389/fpls.2022.1004904

**Published:** 2022-09-29

**Authors:** Hongjuan Zhao, Nan Wang, Hongchun Sun, Lingxiao Zhu, Ke Zhang, Yongjiang Zhang, Jijie Zhu, Anchang Li, Zhiying Bai, Xiaoqing Liu, Hezhong Dong, Liantao Liu, Cundong Li

**Affiliations:** ^1^ State Key Laboratory of North China Crop Improvement and Regulation/Key Laboratory of Crop Growth Regulation of Hebei Province/College of Agronomy, Hebei Agricultural University, Baoding, China; ^2^ College of Mechanical and Electrical Engineering, Hebei Agricultural University, Baoding, China; ^3^ Institute of Cereal and Oil Crops, Hebei Academy of Agriculture and Forestry Sciences, Shijiazhuang, China; ^4^ Cotton Research Center, Shandong Key Lab for Cotton Culture and Physiology, Shandong Academy of Agricultural Sciences, Jinan, China

**Keywords:** rhizopot, high-throughput, growth, image acquisition, phenotyping, plant roots, root hair, root lifespan

## Abstract

Quantitative analysis of root development is becoming a preferred option in assessing the function of hidden underground roots, especially in studying resistance to abiotic stresses. It can be enhanced by acquiring non-destructive phenotypic information on roots, such as rhizotrons. However, it is challenging to develop high-throughput phenotyping equipment for acquiring and analyzing *in situ* root images of root development. In this study, the RhizoPot platform, a high-throughput *in situ* root phenotyping platform integrating plant culture, automatic *in situ* root image acquisition, and image segmentation, was proposed for quantitative analysis of root development. Plants (1-5) were grown in each RhizoPot, and the growth time depended on the type of plant and the experimental requirements. For example, the growth time of cotton was about 110 days. The imaging control software (RhizoAuto) could automatically and non-destructively image the roots of RhizoPot-cultured plants based on the set time and resolution (50-4800 dpi) and obtain high-resolution (>1200 dpi) images in batches. The improved DeepLabv3+ tool was used for batch processing of root images. The roots were automatically segmented and extracted from the background for analysis of information on radical features using conventional root software (WinRhizo and RhizoVision Explorer). Root morphology, root growth rate, and lifespan analysis were conducted using *in situ* root images and segmented images. The platform illustrated the dynamic response characteristics of root phenotypes in cotton. In conclusion, the RhizoPot platform has the characteristics of low cost, high-efficiency, and high-throughput, and thus it can effectively monitor the development of plant roots and realize the quantitative analysis of root phenotypes *in situ*.

## Introduction

The root system is the primary organ for absorbing water and nutrients, and the site for hormone and organic acid synthesis. The root phenotype is closely related to crop yield, quality, and stress resistance (Tracy et al., 2020). Therefore, root phenotype analysis is important for improving crop yield, and stress resistance (Joshi et al., 2017; Rinehart et al., 2022) and thus can promote the breeding of new crop varieties and stress-resistant cultivation measures.

The root phenotype plasticity depends on internal genetic factors and external environmental factors (Lynch, 1995; Gruber et al., 2013; Lynch, 2013), such as temperature (Nagel et al., 2009), moisture, soil structure (Bengough et al., 2011), and soil nutrients (Lambers et al., 2009). The optimal root phenotype in a given environment enables efficient exploration and competition for resources and can survive periods of nutrient or water scarcity (Li et al., 2022b) Therefore, exploring dynamic root phenotypes can further clarify the response mechanism of root phenotypes to the environment and reveal the relationship between root traits and yield for improving crop yield (Tracy et al., 2020).

The underground root system (the hidden half of the plant) is highly complex (Box, 2002; Atkinson et al., 2019). As a result, the study of root phenotypes is highly dependent on research methods. The traditional root phenotype research methods (multiple destructive sampling methods) manually separate the roots from the soil through root drilling method, excavation method (Cheng et al., 2009), clod method (Li et al., 2006), and shovelomics method (Trachsel et al., 2011) for morphological analysis. The destructive sampling root phenotyping method cannot achieve *in situ* root phenotype research. Moreover, it has a large workload and low efficiency and causes root loss during excavation and cleaning (Wasaya et al., 2018), making it difficult to achieve accurate root phenotyping. Therefore, developing a low-cost and high-efficient *in situ* root phenotype research device is necessary for soil substrates and for observing root and root hair phenotypes (Adu et al., 2014).

Non-destructive *in situ* root phenotyping methods enhance the collection of root images (Lynch, 2021) and *in situ* root images (Liu et al., 2020). Digital cameras and scanners are widely used to obtain images for *in situ* root platforms (Jeudy et al., 2016; Wu et al., 2018; (Yuan et al., 2016). Although digital cameras and scanners can collect *in situ* root phenotype images to identify roots, they cannot sufficiently identify root hair phenotypes. Although a microscope can observe root hair phenotypes, microscopy may also change the root hair phenotype during fixation and staining and thus cannot accurately capture root dynamic information (Guo et al., 2009). Therefore, high-throughput and high-resolution image acquisition methods should be developed for *in situ* root phenotype studies.

Root segmentation involves the extraction of roots from the root image via noise reduction, thresholding, and skeletonization. Root segmentation is crucial in *in situ* root phenotyping (Shen et al., 2020) since it enhances root trait analysis. However, root image segmentation and feature extraction are affected by the uneven background of root images, mutual occlusion, and easily movable root tips. Advances in deep learning and other machine learning methods have enhanced root image segmentation.

Convolutional neural networks (CNNs) are widely used in deep learning for image processing. CNNs are a hierarchical structure that performs sequential image filtering operations to transform images from traditional color image inputs into new feature representations. The commonly used image segmentation method relies on a “bottom-up” approach, designing an updated encoder-decoder configuration based on CNNs, combining a CNN with a second inverse CNN to segment images, or extracting the locations of key feature points in the root system (Yasrab et al., 2019). This method filters out pixels other than the root system and groups the root system using multiple filters (Seethepalli et al., 2020). Weka Segmentation (TWS) and RootPainter (Arganda-Carreras et al., 2017b) classify pixels for image segmentation *via* user-input training algorithms. However, they still need further improvement. Therefore, high-throughput *in situ* root image analysis can improve the quality of root image segmentation and *in situ* root research.

In conclusion, dynamic root phenotype analyses can be improved by obtaining high-resolution *in situ* root images, improving the efficiency and accuracy of *in situ* root image segmentation, and quickly obtaining root images (Shen et al., 2020). *In situ* root acquisition and analysis methods are currently in the developmental stage (Li et al., 2022a). Therefore, high-throughput *in situ* image acquisition and root image analysis platforms should be developed to improve dynamic root phenotyping methods. Cotton is one of the most important economic crops with a typical tap root and thus was used in this study to verify the RhizoPot platform, as a method for studying *in situ* root phenotypes. This method can observe changes in root morphology, including the characteristics of changes in fine roots and root hairs, under abiotic stress for quantitative analysis of *in situ* root phenotypes.

This study aimed to develop a general, high-throughput, high-efficiency, and low-cost *in situ* root phenotyping platform (RhizoPot platform) for dynamic root phenotyping analysis. The RhizoPot platform combines RhizoPot, RhizoAuto, DeepLabv3+, and RhizoComp, thus integrating the acquisition, segmentation, and contrast of *in situ* root images, to study the root morphology, root growth rate, and lifespan analysis of roots and root hairs during root development. The performance of the software and hardware of the RhizoPot platform was assessed using two genotypes of cotton (Gossypium hirsutum L.). Therefore, this research may enhance the study of dynamic root phenotyping.

## Materials and methods

### Composition of the RhizoPot platform

**Figure 1:**
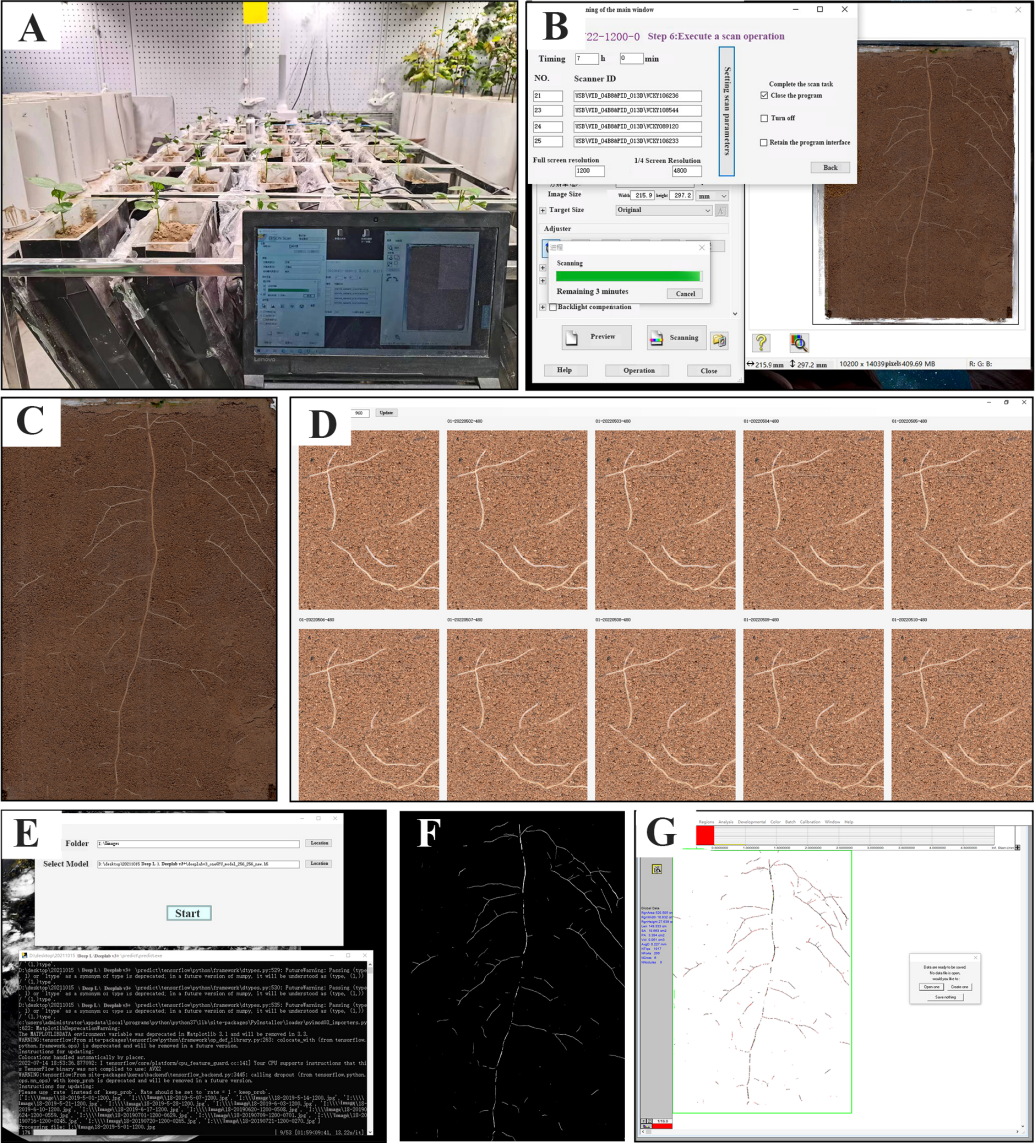
The composition and workflow of the RhizoPot platform for *in situ* root phenotyping. **(A)** RhizoPot in a greenhouse. **(B)** Operation interface of RhizoAuto. **(C)** *In situ* root images from a RhizoAuto-controlled flatbed scanner. **(D)** Image comparison analysis tool RhizoComp. **(E)** Working photo of image segmentation tool DeepLabv3+. **(F)** Rendering of the segmented *in situ* root image. **(G)** The segmented images analyzed with WinRhizo software.

The RhizoPot platform (software and hardware) has four parts: RhizoPot, RhizoAuto, DeepLabv3+, and RhizoComp (**Figure 1**). The diagram below also shows the workflow of the platform. RhizoPot cultivates seedlings, while RhizoAuto collects *in situ* root images. DeepLabv3+ segments the images, which are then analyzed by WinRhizo software to obtain root phenotypes. RhizoComp analyzes *in situ* root images to obtain root lifespan, and the lifespan of root hairs was defined as the number of days from the appearance of root hairs to when the root hairs were distorted.

#### RhizoPot

**Figure 2:**
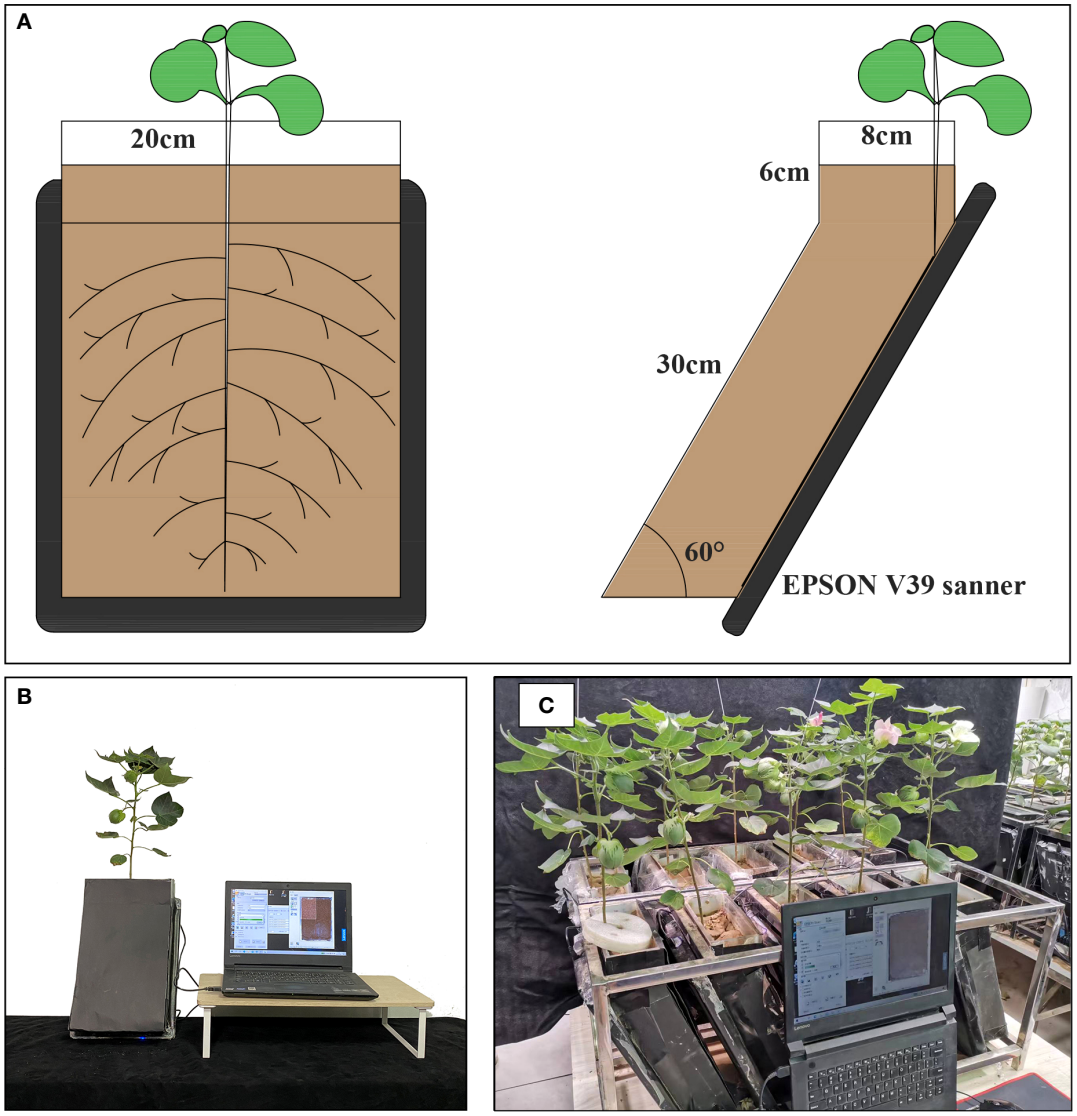
RhizoPot’s single cross-cuts **(A)**, single entity photos **(B)**, and batch work drawings **(C)**.

RhizoPot is a plant culture container consisting of an acrylic plate and a scanner (Epson Perfection Version 39, Suwa, Japan), with soil as the substrate to simulate the field growth environment (**Figure 2A**). In this experiment, the RhizoPot was tilted at 60°. The panel of the flatbed scanner replaced the acrylic plate on one side, and it was placed in close proximity to the culture substrate to obtain a high standard image of the root system (**Figures 2A, B**). The inclined culture vessel enables more roots to come into contact with the panel using the scanner. It also facilitates the placement of RhizoPot, improves space utilization, and facilitates high-throughput assays (**Figure 2C**). The dimensions of the RhizoPot are 20 W x 8.5 D x 34 H cm^3^ (W, D, and H represent width, depth, and height, respectively). The outer wall of the RhizoPot is covered with a layer of black cardboard to prevent root exposure to light. About 1-5 plants were grown in each root culture container (sowing position; about 1 cm away from the side of the scanner panel).

#### RhizoAuto

**Figure 3:**
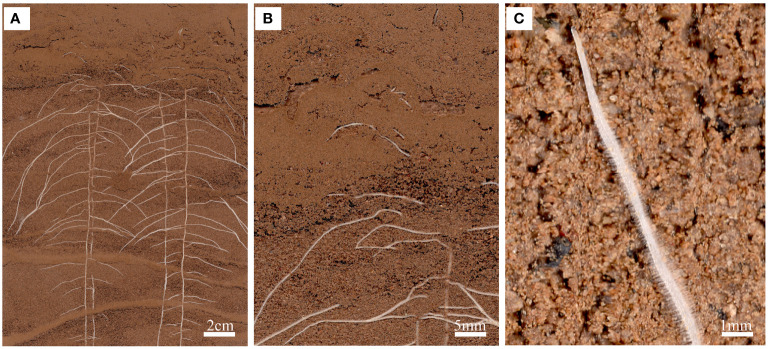
An *in situ* root phenotype with a cluttered scene. **(A)** A sample input image of the root system of a cotton seedling growing on RhizoPot. **(B)** The scene with cluttered colors represents the difficulty associated with image segmentation. **(C)** Visible root hairs and root tips.

RhizoAuto controls the RhizoPot scanner to automatically capture high-resolution root images. It is programmed in Java and sends the image capture command to the working scanner using the TWAIN module (http://TWAINmodule.sourceforge.net), which is then transferred to the computer. Each RhizoAuto can simultaneously control 10 RhizoPot flatbed scanners and run based on the set acquisition time, resolution, and file naming method. RhizoAuto first acquires a 1200 dpi resolution image, then a 4800 dpi resolution image. Herein, the 1200 dpi images were used for analyzing root morphological characteristics and lifespan, while the 4800 dpi images were used to analyze root hair phenotype and lifespan (**Figure 3**).

#### DeepLabv3+

DeepLab v3+ automatically segments roots from *in situ* root images with cluttered scenes into root images with a single background. It is based on deep learning, adopting the encoder-decoder structure based on CNN (Shen et al., 2020). It introduces a sub-pixel convolution method and an efficient upsampling method based on deep learning.

The convolution operation extracts the target features and obtains low-resolution feature maps. The sub-pixel convolution-based upsampling process can effectively improve image quality (Zhao et al., 2019). Therefore, subpixel convolution during root pixel segmentation can prevent pixel loss after standard DeepLabv3+ sampling, improve the segmentation accuracy of small and indistinguishable root trajectories, and further enhance the accuracy and integrity of the root system after segmentation. Image segmentation makes the root system white with a black background, which is suitable for WinRHIZO (Regent Instruments, Inc., Quebec City, Canada) analysis to obtain conventional root system indicators, including total root length (RL, cm), average root diameter (AD, mm) and total root surface area (RSA, cm^2^).

Herein, WinRHIZO Tron MF (Regent Instruments, Inc., Quebec City, Canada) was used for manual segmentation of *in situ* root images. The operator manually tracked and marked all roots using a mouse, segmented all roots, and then obtained root indicators based on the segmentation results. The effect and efficiency of the two root image segmentation methods were then compared to verify the efficacy of the DeepLabv3+.

### Dynamic root phenotypes

The root morphological indexes were obtained after segmenting the regularly collected 1200 dpi root images. The change characteristics of root phenotype, such as root growth rate, root diameter change rate, and net root growth per unit volume, were calculated based on the time of *in situ* root image collection. This can enhance research on the growth rate and change characteristics of the root system in response to environmental changes (Suwanchaikasem et al., 2022).

The characteristics of root morphological changes were calculated as follows:

RLD = RL/(A×DOF)
RLD_NGR_=(RLD_n_-RLD_n-1_)/d

Where RLD represents the root length density (Salim et al., 2021); L, A, and DOF represent the root length observed in RhizoPot frames (m), observed RhizoPot frame area, and the depth-of-soil (2.5 mm), respectively (Johnson et al., 2001); RLD_NGR,_ RLD_n,_ and d represent net root growth per unit volume, root length density on the specified date after sowing, and time, respectively.

### Root hair phenotype

Root hair length and density were measured using *in situ* root images (4800 dpi) *via* Adobe Photoshop CC 2019 (Adobe, San Jose, CA, USA). Briefly, “Histogram”, “Measurement Record” and “Information” were selected in the menu bar to facilitate statistics and calculations. The 4800 dpi root image was opened with the ruler tool, and a distance of 1 cm was selected on the photo, then measured by selecting “Record”. The number of pixels corresponding to each centimeter was obtained, and the root segment with root hairs was located. Five root hairs were randomly selected for measurement, and the number of pixels corresponding to the length of the root hair was calculated (Xiao et al., 2020). The length and density of root hair were then determined. ImageJ (https://imagej.net) was used to complete the work instead of Adobe Photoshop.

### RhizoComp for analysis of root system and root hair longevity

RhizoComp, a root image comparison tool based on C+, can simultaneously perform operations on ten *in-situ* root image by zooming and moving. Herein, *in situ* root images of different time series were imported into RhizoComp. The entire process from emergence to senescence of a root was visualized by synchronizing zoom in and move. The lifespan of roots or root hairs was then determined using the time interval between the two images (Parent and Tardieu, 2012). Newly emerging roots are white with root tips, while senescent roots are dark brown or black with the distinctly folded epidermis (Hendrick and Pregitzer, 1992). This tool was also used to analyze root hair lifespan. The 4800 dpi root image was enlarged to the root system until the root hair was visible. The time of appearance and senescence of root hair in this area was searched, and its lifespan was judged based on the time interval. Root hair senescence was defined as senescence based on morphological changes when the root hair is twisted and bent (Hendrick and Pregitzer, 1992).

### Validation of the RhizoPot platform

Validation was conducted in 2021 at the Hebei Agricultural University (38° 85′ N, 115° 30′ E), Hebei Province, China, using two commercial cotton (*Gossypium hirsutum* L) cultivars “K836” and “K837”. Sowing was performed 1 cm from the RhizoPot scanner panel, and the two seeds were sown 8 cm apart. The experiment had ten replicates. The plants were grown in a smart greenhouse under day/night temperatures of 28/25 °C, 14/10 h (light/dark) photoperiod, and a light intensity of 600 µmol/m^2^/s^1^. *In situ* root images (1200 dpi and 4800 dpi) were automatically acquired using RhizoAuto every day at 8 am for 110 d. The *in situ* root images were processed using DeepLabv3+ and RhizoComp to analyze the root growth rate, diameter change rate, net growth per unit volume, root lifespan, root length density, and lifespan of root hairs.

### Data statistical analysis

One-way ANOVA was used to determine significant differences among the tested genotypes for each trait *via* SPSS Statistics 20 (IBM, USA). GraphPad Prism 8.0.2 (CA, USA) and Origin 2022b (OriginLab, USA) were used to plot figures. Statistical significance was set at P ≤ 0.05.

## Results

### A high-throughput integrated platform for plant culture and *in situ* root studies

**Figure 4:**
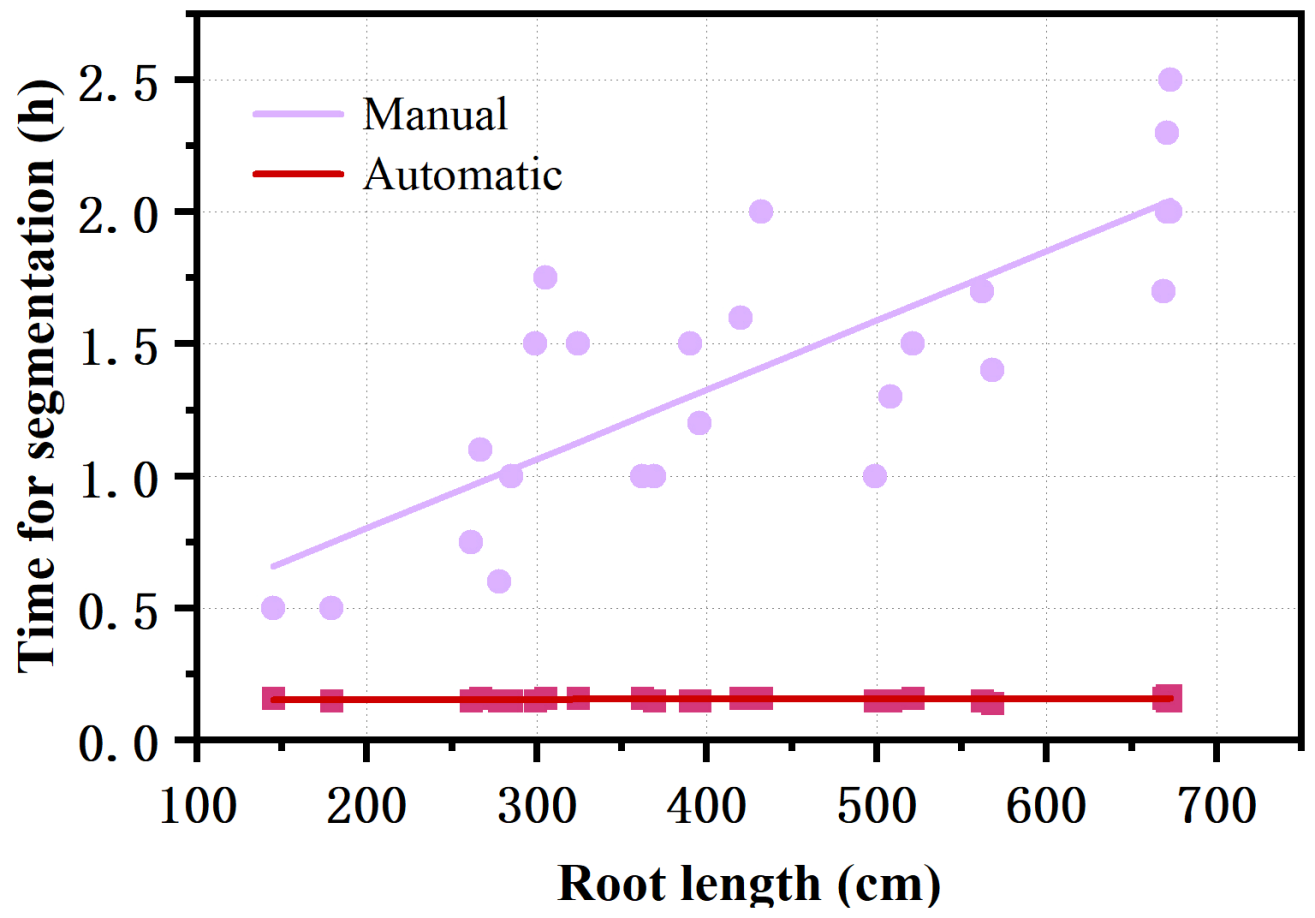
Comparison of the efficiency of automatic segmentation and manual segmentation of images with different root lengths.

The RhizoPot platform cultivated cotton to the boll opening period using soil or mixed substrates. The growth period was about 110 d (not limited to this number of days). The flatbed scanner was controlled using AutoScan to efficiently and automatically collect *in situ* root images in batches. The 1200 dpi and 4800 dpi root images were acquired at about 3 min/image and 10 min/image, respectively. The speed of *in situ* root image segmentation using DeepLabv3+ (Automatic segmentation) was about 10 min/image, significantly higher than manual segmentation’s efficiency (**Figure 4**). The automatic segmentation achieved batch and automatic root image segmentation. The daily changes in root phenotypic characteristics were compared, and the dynamic root phenotype and root lifespan were calculated.

The approximate cost of the RhizoPot platform, including each component, is detailed in **Table 1**. The total cost for the culture vessel, imaging sensor, and connection accessories was about $237 but could vary depending on labor input or bulk consumables used.

**Table 1:** Details and costs of individual components in the RhizoPot platform.

No.	Component	Size	Approximate cost per unit
1	Epson Scanners	Epson Perfection Version 39	$192
2	Transparent acrylic plastic	0.5 m^2^	$5
3	Black cardboard	0.5 m^2^	$2
4	Sealing glass glue	500 ml	$6
5	USB HUB	With power cord	$2
6	Labor cost	Cutting, splicing and sealing, etc.	$30
		Total	$237

### Validation using two cotton varieties

#### The effect of DeepLabv3+ segmenting *in situ* root images

**Figure 5:**
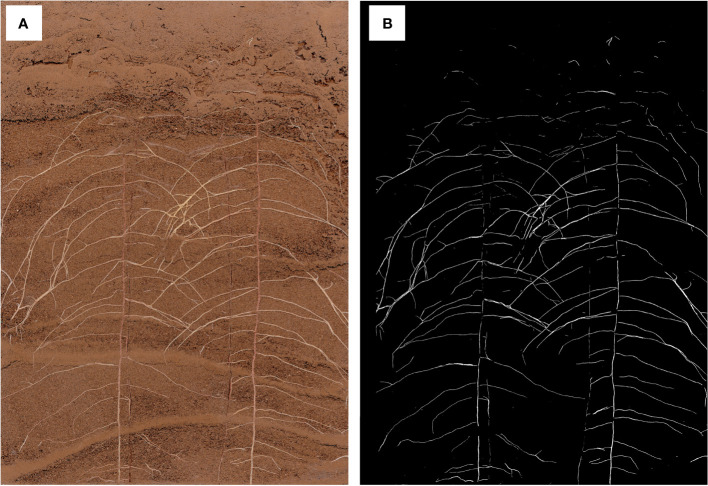
*In situ* root image **(A)** and image after segmentation with DeepLabv3+ **(B)**.

The segmentation effect of DeepLabv3+ on *in situ* root images was evaluated as follows: The root length, root surface area, root volume, and mean root diameter between DeepLabv3+ and manually segmented root images were compared using the collected cotton root images (**Figures 5**; **S1**). The DeepLabv3+ segmentation had a higher correlation with the manual segmentation, with root length having the highest correlation (R^2^ of 0.91) (**Figure S1**).

#### Dynamic root phenotypic traits of cotton

**Figure 6:**
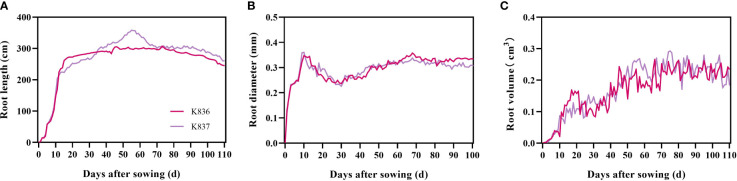
Dynamic characteristics of root phenotypes of two cotton varieties. **(A)** Root length, **(B)** root diameter, and **(C)** root volume.

The root length of the two cotton varieties significantly increased from 1 d to 7 d, mainly due to the appearance and elongation of lateral roots (**Figure 6**). The root length then slowly increased (K836 and K837 reached the maximum at 62 d (302.52 cm) and 55 d (358.64 cm), respectively) (**Figure 6A**). The root diameter decreased from 10 d to 30 d due to the relatively large diameter of the main root at the initial stage, which decreased the average root diameter after lateral roots appeared in the early stage (**Figure 6B**). The root diameter rapidly increased at 30 d, then decreased at 68 d due to the senescence of the root system and the reduction of the thickness of the cortex. The root volume increased through the growth. However, the growth rate was faster in the early stage than in the later stage due to the rapid growth of primary and secondary lateral roots (**Figure 6C**).

**Figure 7:**
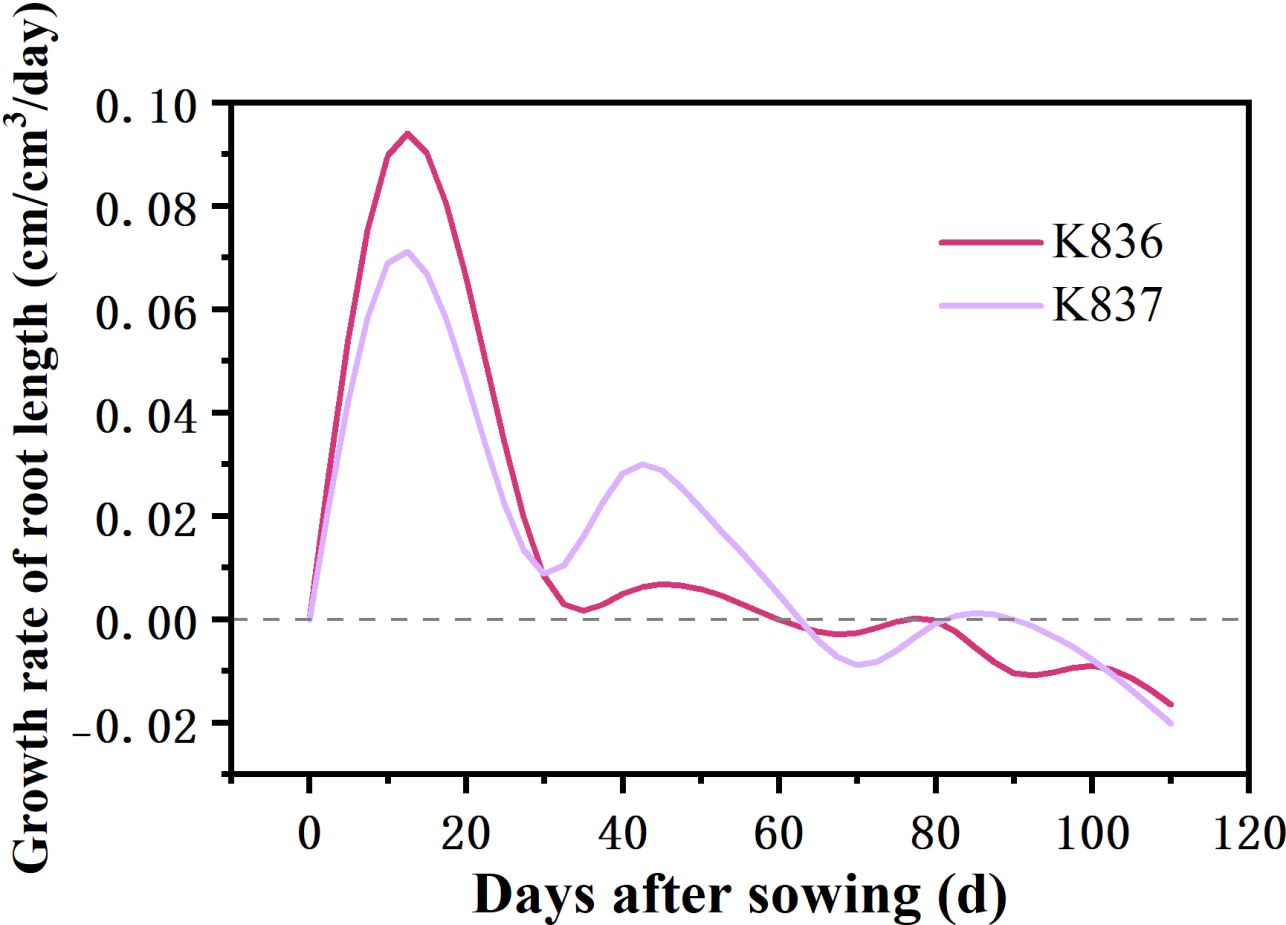
Net growth rate of root length per unit volume (cm/cm^3^/day) of two cotton varieties (RLD_NGR_).

The net growth rate of root length per unit volume (RLD_NGR_) rapidly increased in the early stage, reaching the maximum at 10 d, then decreased to a negative value after 60 d (**Figure 7**). Root senescence and elongation growth simultaneously occurred in the later period of cotton growth. However, the trend of senescence was greater than that of elongation growth.

**Figure 8:**
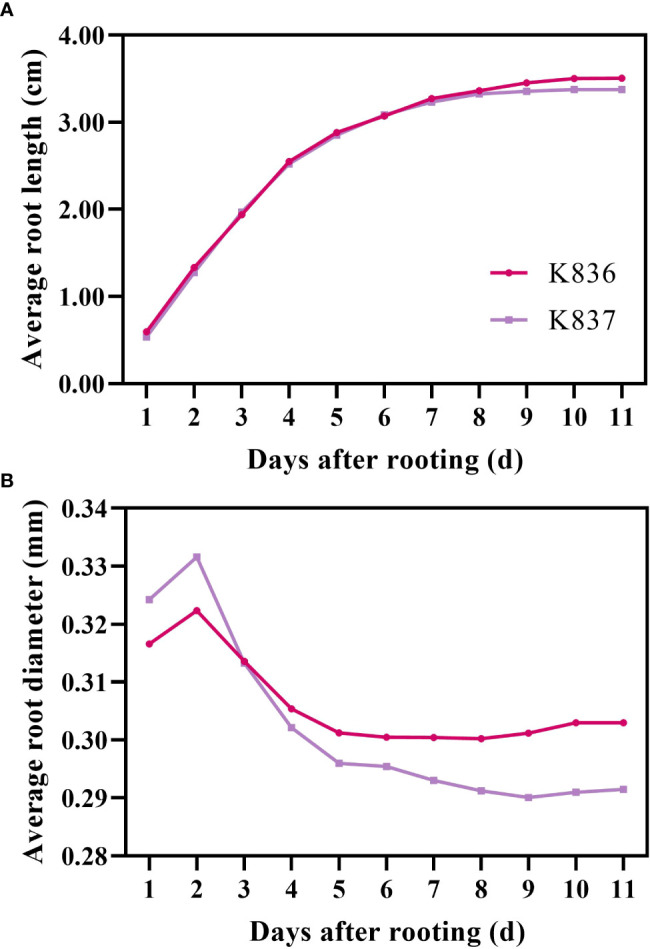
Growth rate of average root length and root diameter. **(A)** Average root length and **(B)** average root diameter. The primary lateral roots of K836 and K837 were 116 and 92, respectively.

The growth dynamics of a single root system were determined using root system images collected by the RhizoPot platform (**Figure 8**). The root system length significantly changed in the first 7 d, then gradually changed, becoming stable after 10 d. The root diameter first increased, then decreased (**Figure 8B**). The lateral root diameter of K836 and K837 did not significantly change at 8 d and after 10 d of rooting, respectively.

#### Phenotypic traits of root hairs in cotton

**Figure 9:**
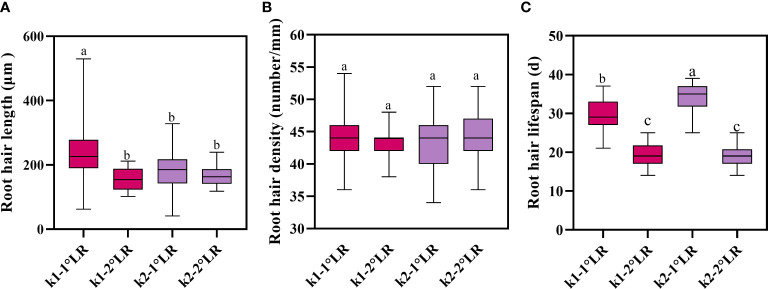
Root hair traits of cotton. **(A)** Average root hair length, **(B)** average root hair density, and **(C)** average root hair lifespan. K1-1°LR, the primary lateral roots of K836; K1-2°LR, the secondary lateral roots of K836; K2-1°LR, the primary lateral roots of K837, K2-2°LR, the secondary lateral roots of K837. Different letters indicate significant differences between treatments (p<0.05).

The root hair lengths of primary and secondary lateral roots of K836 cultivar were significantly different (**Figure 9A**). In contrast, the root hair length of primary and secondary lateral roots of K837 cultivar was not significantly different. The root hair density was not significantly different between the two varieties (**Figure 9B**). Moreover, root hair length did not significantly affect root hair density.

#### Lifespan of roots and root hairs of cotton

The average lifespan of primary lateral root hairs of K836 and K837 was 30 d and 34 d, respectively (**Figure 9C**). The average lifespan of secondary lateral root hairs of K836 and K837 was shorter than that of primary lateral root hairs by 11 d and 15 d, respectively.

**Figure 10:**
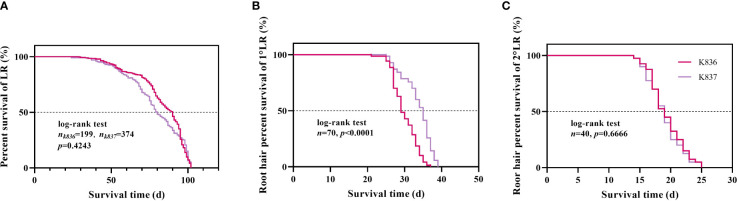
Survival analysis of lateral roots and root hair. **(A)** Lateral roots. **(B)** Root hair of the primary lateral roots. **(C)** Root hair of the secondary lateral roots.

Survival analysis showed that the median lifespan of K836 lateral roots was longer than that of K837 lateral roots by 10 d (**Figure 10A**). However, the median lifespan of K836 primary lateral root hairs was shorter than that of K837 primary lateral root hairs by 6 d (**Figure 10B**). Besides, the median lifespan of secondary lateral root hairs was not significantly different between the two cultivars (**Figure 10C**).

**Figure 11:**
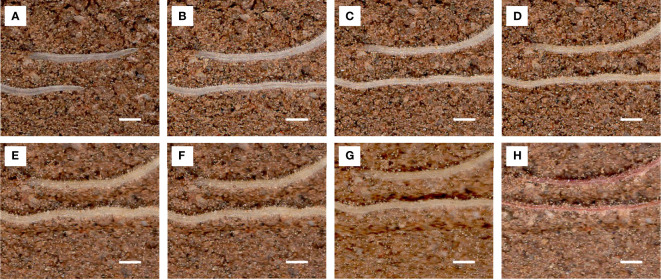
Images of the same root area at different time points used to calculate root lifespan. Scale bar, 1 mm. Root pictures for different days: **(A)** 1 d, **(B)** 10 d, **(C)** 20 d, **(D)** 30 d, **(E)** 40 d, **(F)** 50 d, **(G)** 60 d, and **(H)** 70 d (dead roots).

Lateral root lifespan was defined as the number of days between the appearance of the lateral root and the time when its appearance turned brown. The entire process of root from emergence to distortion and deformation was observed (**Figure 11**).

## Discussion

### RhizoPot platform enhances the efficiency of *in situ* root imaging and analysis

In this study, the developed RhizoPot platform had the following characteristics; low-cost, high-efficiency, and high-throughput. Cameras (Bates and Lynch, 1996), monochrome vision cameras (Seethepalli et al., 2020), and industrial digital lenses (Liu et al., 2020) are the commonly used imaging equipment for the *in situ* root imaging platform. However, these types of equipment are expensive and not suitable for large-scale use. The developed platform had a low-cost flatbed scanner, which can obtain images at a wide range of resolutions, providing the basis for *in situ* root phenotypic analysis. Moreover, the 4800 dpi resolution image can be used to analyze root hair phenotypes. The vessel maximizes the representation of actual root characteristics of plants using soil as the culture medium. Furthermore, this platform improves the root image by making the root system grow directly on the scanner. Although the platform can obtain high-resolution images, the 1200 dpi and 4800 dpi root images take 3 and 10 min, respectively. Furthermore, RhizoPot can automatically acquire each *in situ* root image without human intervention, thus significantly improving efficiency compared with manual operations. In summary, the high-resolution root images obtained through this platform provide a basis for root phenotype studies and plant and microorganism interactions (Jeudy et al., 2016).

This platform can also automatically acquire root images. The computer obtains root images through AutoScan control scanner. Each computer can connect and control 10 scanners. Automatic image acquisition is completed by setting relevant parameters of AutoScan (scanning time and resolution). This platform is more efficient and flexible than individual culture vessels, such as RhizoTubes (Jeudy et al., 2016), RhizoChamber-Monitor (Wu et al., 2018), and root-TRAPR (Suwanchaikasem et al., 2022).

In addition, a deep learning-based image segmentation software DeepLabv3+ was developed. DeepLabv3+ combines the advantages of an encoder-decoder architecture and atrous spatial pyramid pooling (ASPP). DeepLabv3+ can capture rich associated information from plant root images across various resolutions, identifying and segmenting clear root trajectories. The results of DeepLabv3+ were highly correlated with the results of the conventional manual tracing method with WinRHIZO Tron MF (**Figure 4**). DeepLabv3+ can also complete image segmentation in batches while maintaining the integrity of the primary root and the continuity of the fine root edge contour without user interaction. DeepLabv3+ can automatically, efficiently, and accurately complete the segmentation of the root image, thus facilitating the acquisition of root indicators.

Meanwhile, RhizoComp was also developed to observe the root senescence process. RhizoComp can simultaneously observe ten high-resolution root images by zooming and moving the images synchronously, thus enabling continuous observations of the aging process of one or more roots. Compared with the traditional method of collecting *ex vivo* roots and observing through microscopy, this method significantly improves the efficiency and accuracy for root lifespan study.

### Quantitative analysis of root development with RhizoPot platform

High-quality root images are important for root phenotyping analysis (Jeudy et al., 2016). Analyzing high-resolution *in situ* root images based on time series enhances studying of dynamic root phenotype characteristics. As a result, it facilitates the analysis of changes in root phenotype between genotypes or species in response to environmental changes (Niu et al., 2018). Root length growth rate, root diameter growth rate, root hair lifespan can be calculated using the root phenotype change parameters in a specific time (Xiao et al., 2020; Zhang et al., 2021; Zhu et al., 2022), thus promoting the study of the root system response to adversity. In this study, the high-resolution root images obtained by the RhizoPot platform were used to study the dynamic characteristics of lateral root phenotype (Zhu et al., 2022). The platform could better simulate the root development of the field conditions with high-throughput and efficiency compared with agar culture-based phenotypic methods (Zhu et al., 2022).

Several *in situ* root phenotyping platforms, such as RhizoChamber-Monitor (Wu et al., 2018) and RhizoTubes (Jeudy et al., 2016), have been developed for root image acquisition with high-efficiency and high-throughput and in a non-destructive manner. However, these platforms adopt the paper-based culture, which limits the representation of actual root characteristics of plants grown in soils, and thus can only be used to examine the root system of seedlings. RhizoPot simulates the field growth environment using soil or mixed culture media and thus can meet the need of the entire growth period of field crops (110 d). Yuan et al. (2016) developed a Brassica–rhizotron system with soil as the culture medium (Vessel volume; about 118 L). This approach was not suitable for automated operation, and the image quality was not high enough since the scanner panel was not in direct contact with the culture medium. In RhizoPot, a flatbed scanner is usually used as an imaging device, and it is in direct contact with the culture medium, thus improving the image quality. However, using a scanner as a panel has a limitation since the panel may be deformed by the gravitational pressure of the culture medium, resulting in failure of the scanning sensor and failure of the scanner. Therefore, related research is needed to improve this panel.

### Application and Expansion of RhizoPot Platform

The RhizoPot platform is suitable for studying root development under various abiotic stresses, such as high temperature, drought, salinity, heavy metal, and oxidative stress. In this study, this platform was used to assess the characteristics of dynamic root phenotypes of two cotton varieties. The root growth rate, lateral root traits, and lateral root and root hair lifespan were also analyzed. A high degree of correlation was achieved (R^2^> 0.8) by comparing the results with the traditional method results, reflecting the advantages of the platform.

The platform can also be used to evaluate root structure of other species, such as legumes (Burridge et al., 2020). It can also be used to study phenotypic characteristics of plant roots under biological stress, such as the rate and law of change of root spots or the development process of roots in response to underground pests. Moreover, the platform can be used to study plant and microorganism interactions.

## Conclusion

In this study, the RhizoPot platform was developed to analyze phenotypic characteristics during root development. RhizoPot is a high-throughput *in situ* root phenotyping platform that integrates plant culture, automated *in situ* root image acquisition, and image segmentation for quantitative analysis of root development. Herein, this platform was used to illustrate the dynamic response characteristics of root phenotypes in cotton. The platform was also used to explore dynamic root phenotypic traits, such as net growth rate of root length and lifespan, during cotton root development and verified root image segmentation efficiency, etc. The root image segmentation tool was effective and accurate, significantly outperforming manual segmentation, especially in terms of root length. *In situ* root images and segmented images can be used for root morphology, root growth rate, and lifespan analyses. In summary, the RhizoPot platform was low-cost and had high-efficiency and throughput. Therefore, it can be used to monitor the dynamic root phenotype characteristics during plant development and realize the quantitative analysis of root phenotype *in situ*.

## Data availability statement

The raw data supporting the conclusions of this article will be made available by the authors, without undue reservation.

## Author contributions

LL, HD, and CL initiated and designed the research. HZ, HS, XL, and LZ performed the experiments and collected the data. NW, LZ, and AL wrote the code and tested the methods. HZ, HS, KZ, YZ, JZ, and ZB analyzed the data and wrote the manuscript. All authors contributed to the article and approved the submitted version.

## Funding

This study was supported by the National Natural Science Foundation of China (No. 31871569 and 32172120), Natural Science Foundation of Hebei Province (No. C2020204066), Graduate Innovation Funding Project of Hebei Province (CXZZBS2020089), and the Modern System of Agricultural Technology in Hebei Province (No. HBCT2018040201).

## Conflict of interest

The authors declare that the research was conducted in the absence of any commercial or financial relationships that could be construed as a potential conflict of interest.

## Publisher’s note

All claims expressed in this article are solely those of the authors and do not necessarily represent those of their affiliated organizations, or those of the publisher, the editors and the reviewers. Any product that may be evaluated in this article, or claim that may be made by its manufacturer, is not guaranteed or endorsed by the publisher.

## Supplementary material

Supplementary Figure 1 Comparison of root phenotypic traits between automatically segmented (DeepLabv3+) and manually segmented *in situ* root images. **(A)** Root length, **(B)** average root diameter, **(C)** root surface area, and **(D)** root volume estimation results.
