# Supplementary figures and images for: RhizoPot platform: A high-throughput in situ root phenotyping platform with integrated hardware and software
Source: Front Plant Sci. 2022 Sep 29;13:1004904. doi: 10.3389/fpls.2022.1004904 (PMC9558169; doi:10.3389/fpls.2022.1004904)

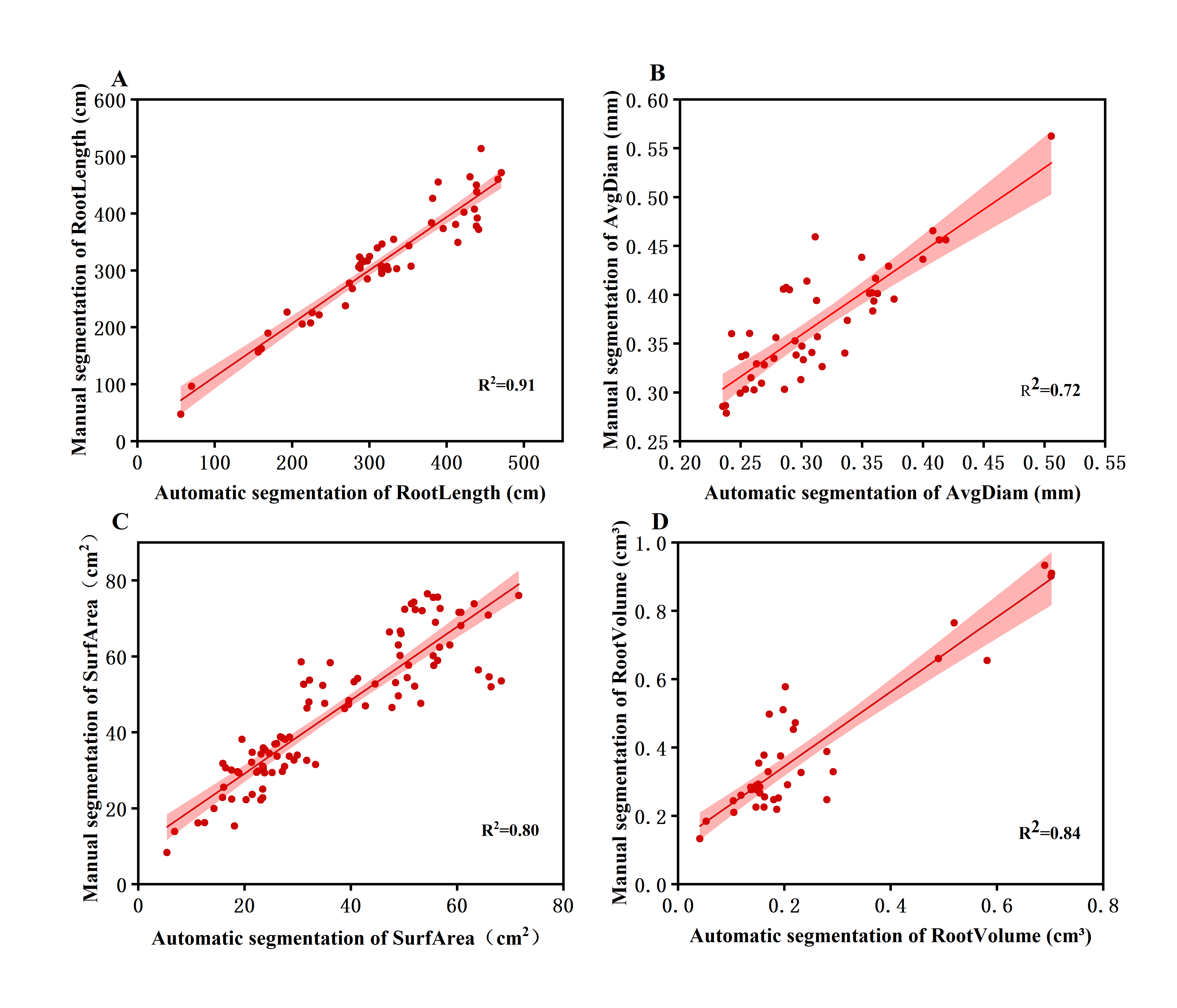

Supplement: Supplementary Figure 1 — Comparison of root phenotypic traits between automatically segmented (DeepLabv3+) and manually segmented in situ root images. (A) Root length, (B) average root diameter, (C) root surface area, and (D) root volume estimation results. [file Image_1.jpeg]
